# Supplementary material for: Selective photoelectrochemical oxidation of glucose to glucaric acid by single atom Pt decorated defective TiO2
Source: Nat Commun. 2023 Jan 10;14:142. doi: 10.1038/s41467-023-35875-9 (PMC9831984; doi:10.1038/s41467-023-35875-9)
Supplement: Supplementary file 1 — Supplementary Information [file 41467_2023_35875_MOESM1_ESM.pdf]

SUPPLEMENTARY INFORMATION

Selective Photoelectrochemical Oxidation of Glucose to Glucaric Acid by Single-Atom Pt Decorated Defective TiO_2

Zhangliu Tian[1,#,*], Yumin Da[1,2,#], Meng Wang[1,2], Xinyu, Dou[1], Xinhang Cui[1], Jie Chen[2,3], Rui Jiang[4], Shibo Xi[5], Baihua Cui[1,2], Yani Luo[1,2], Haotian Yang[1,2], Yu Long[1,2], Yukun Xiao[1,2], and Wei Chen[1,2,3,6,*]

[1] Department of Chemistry, National University of Singapore, 3 Science Drive 3, Singapore, 117543 Singapore.

[2] Joint School of National University of Singapore and Tianjin University, International Campus of Tianjin University, Binhai New City, Fuzhou, 350207, China

[3] Department of Physics, National University of Singapore, 2 Science Drive 3, Singapore, 117542 Singapore.

[4] School of Materials Science and Engineering, Tianjin University, Tianjin 300072, China

[5] Institute of Sustainability for Chemicals, Energy and Environment, Agency for Science, Technology and Research (A*STAR), 1 Pesek Road, Jurong Island, Singapore 627833, Singapore

[6] Centre for Hydrogen Innovations, National University of Singapore (Singapore), E8, 1 Engineering Drive 3, Singapore 117580

[#] These authors contributed equally: Zhangliu Tian, Yumin Da

Email: tianzl@nus.edu.sg; phycw@nus.edu.sg

Supplementary Methods

Characterization of the samples: X-ray diffraction (XRD, Bruker D8 Advance), UV-Vis-NIR spectrometer (Hitachi U4100), X-ray photoelectron spectroscopy (XPS, ESCALAB 250Xi) with Al K*a* X-ray as the excitation source, field emission scanning electron microscopy (FE-SEM, JEOL JSM6700F), transmission electron microscopy (TEM, FEI Titan 80-300, operated at 200 kV), high-angle annular dark-field scanning transmission electron microscope (HAADF-STEM: JEM-ARM200F, 200kV), and Perkin Elmer Avio 500 Inductively Coupled Plasma-Optical Emission Spectrometer (ICP-OES) were conducted to investigate the microstructure and composition of the samples. X-ray absorption fine structure (XAFS) spectra of Pt L3-edge were performed at the X-ray absorption fine structure for catalysis (XAFCA) beamline of the Singapore Synchrotron Light Source (SSLS), Singapore.

Photoelectrochemical (PEC) measurements: The PEC performance of the samples was measured in a three-electrode system with an electrochemical workstation (CHI 760E) under AM 1.5G simulated sunlight of 100 mW cm^{-2}. The simulated solar illumination was obtained from a 300 W Xenon lamp (Microsolar 300; Beijing Perfectlight) with an AM 1.5G filter (100 mW cm^{-2}). Samples on FTO substrates were used directly as the working electrode, with a Pt wire and an Ag/AgCl (KCl saturated) electrode as counter and reference electrodes respectively. The active areas for the working and counter electrodes are 1.5×2.1 cm^2 and 1 cm^2, respectively. All the samples were illuminated through the sample side (front-side illumination). The PEC performance was recorded in an H-type PEC cell, in which 30 ml cathode and 30 ml

anode chambers were separated by an anion-exchange membrane (Fumasep FAA-3-PK-130). The anode chamber was filled with 20 ml, 1 M KOH solution dissolved with 10 mM glucose and the cathode chamber electrolyte was filled with 20 ml, 1 M KOH solution. The reaction temperature for all photoanodes is maintained at 20 °C. Mott-Schottky plots were derived from impedance-potential tests conducted at a frequency of 1 kHz in dark. Intensity-modulated photocurrent spectroscopy (IMPS), small perturbation transient photocurrent measurements, and incident photon-to-current conversion efficiency (IPCE) were recorded by the Zahner Zennium C-IMPS system. The IPCE value was measured at 0.6 V_{RHE} under various monochromatic light irradiation and calculated by Supplementary Equation (1):

$$\mathrm{IPCE} = \frac{J(\mathrm{mA\ cm^{-2}}) \times 1239.8(\mathrm{V\ nm})}{\lambda(\mathrm{nm}) \times P_{\mathrm{mono}}(\mathrm{mW\ cm^{-2}})} \times 100\% \quad (1)$$

where J is the photocurrent density, λ is the wavelength of the incident light, and P_{mono} is the illumination intensity at different wavelengths. The incident photo-to-GLA conversion efficiency of the Pt/def-TiO_2 photoanode at 0.6 V_{RHE} was calculated by Supplementary Equation (2):

$$\text{Incident photo} - \text{to} - \text{GLA conversion efficiency} = \text{IPCE} \times f_{\text{GLA}} \quad (2)$$

where f_{GLA} is the faradaic efficiency for GLA production.

Quantification analysis of the reaction products: The glucose and its products from the PEC cell were analyzed by a Shimadzu LC-20AT high-performance liquid chromatography (HPLC) equipped with a refractive index detector. 5 mM H_2SO_4 at a flow rate of 0.6 mL min^{-1} was used as the mobile phase. In each analysis, ten times diluted electrolyte withdrawn from the PEC cell was injected directly into a BioRad

Aminex 87H column with a column temperature of 60 °C. The glucose and its products were identified and quantification analyzed by comparing their retention times in the chromatograms with those of the standard solution. Gaseous reduction products were analyzed using an online gas chromatograph with Ar as the carrier gas (GC, Shimadzu 2014). The conversion (X_G), yield ($Y_{proudcts}$), and selectivity ($S_{proudcts}$) were calculated using Supplementary Equation (3-5):

$$X_{\mathrm{G}} = \frac{n_{\mathrm{Gi}} - n_{\mathrm{G0}}}{n_{\mathrm{G0}}} \times 100\% \quad (3)$$

$$Y_{\mathrm{products}} = \frac{n_{\mathrm{products}}}{n_{\mathrm{G0}}} \times 100\% \quad (4)$$

$$\mathrm{S}_{\mathrm{products}} = \frac{n_{\mathrm{products}}}{n_{\mathrm{Gi}} - n_{\mathrm{G0}}} \times \frac{\mu}{\varphi} \times 100\% \quad (5)$$

where n_{G0}, n_{Gi}, and $n_{products,}$ are the initial mole number of glucose, the residual number of glucose, and the generated mole number of the products. The μ and φ represent the stoichiometric coefficients of the reaction.

The faradaic efficiencies ($f_{products}$) for the products were calculated by Supplementary Equation (6):

$$f_{\mathrm{products}} = \frac{n_{\mathrm{products}} \times \mathrm{m} \times \mathrm{F}}{\mathrm{Q}} \times 100\% \quad (6)$$

where m represents the quantities of charge required for the generation of one product, F is Faraday's constant (96485.33 C mol^{-1}), and Q is the total charge.

2D-HMBC NMR measurements: To obtain the 2D-HMBC NMR Spectra, we use deuteroxide instead of deionized water as the solvent. 1 M KOH deuteroxide solution with 10 mM glucose was used as the electrolyte. The Pt/def-TiO_2 NRAs were used as the photoanodes to conduct the PEC glucose oxidation at 0.6 V_{RHE} in the deuteroxide solution, under AM 1.5 simulated sunlight irradiation for different times (0-5.5 h). 0.5

ml anolyte was syringed out from the cell at a specific reaction time (1, 3, 5.5 h), and the anolyte was adjusted to acidity with H_2SO_4. Then the obtained acid electrolyte was mixed with deuterated dimethyl sulfoxide (DMSO) as the reference. Subsequently, the final electrolyte was transferred to an NMR sample tube to do the 2D-HMBC NMR analysis by using Bruker AVANCE NEO 500 NMR spectrometer with a water suppression method.

EPR trapping measurements： 5, 5-dimethyl-1-pyrroline N-oxide (DMPO) was used to trap the generated hydroxyl radical in the reaction system at room temperature. Briefly, 0.5 mg Pt/def-TiO_2 powders collected from the FTO substrates were dispersed in 2 mL of 1 M KOH solution, and then 50 μL DMPO was added into the solution. The mixture was then filled with Ar and sonicated for 1 min. After the sonication, the solution was irradiated by a 300 W xenon lamp with an AM 1.5 filter for 30 s. The resulting solution was subjected to analysis by using a JEOL (FA200) ESR Spectrometer.

In-situ Fourier transform infrared spectroscopy (FTIR) measurements： 5 mg Pt/def-TiO_2 NRAs detached from the FTO substrates and 80 μL 5 wt % Nafion solution were dispersed in 2 mL of 4:1 v/v water/ethanol by sonication for 1 h to form a homogeneous ink. 30 μl ink dispersion was dropped onto the central area (confined by an O-ring with Φ=8 mm) of an Au film chemically deposited on the basal plane of a hemicylindrical Si prism. The Si prism as working electrode was assembled in a spectroelectrochemical cell with Pt wire as counter electrode, Ag/AgCl electrode as reference electrode, and 1 M KOH with 10 mM glucose as electrolytes. In-situ FTIR

spectra were measured on a Nicolet iS50 spectrometer, equipped with an MCT cryogenic detector with a resolution of 4 cm^{-1} each single-beam spectrum was an average of 200 scans. A CHI 760e electrochemical workstation (Shanghai CH Instruments, Inc.) was used for potential control. A light-emitting diode (LED) ultraviolet light (365 nm) was used as the light source for the PEC experiments.

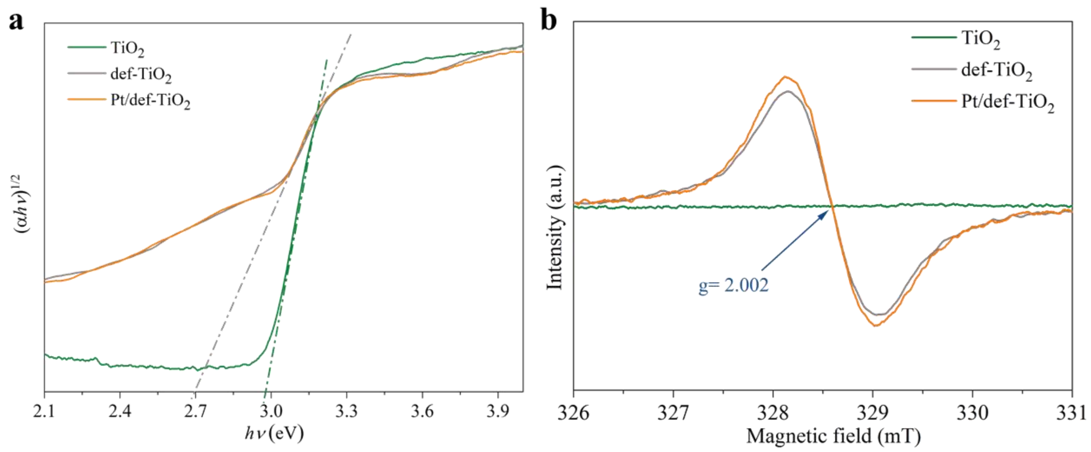

Supplementary Fig. 1 Band gap and oxygen vacancies of NRAs. (a) Tauc plots and (b) ESR spectra of the TiO_2, def-TiO_2, and Pt/def-TiO_2 NRAs.

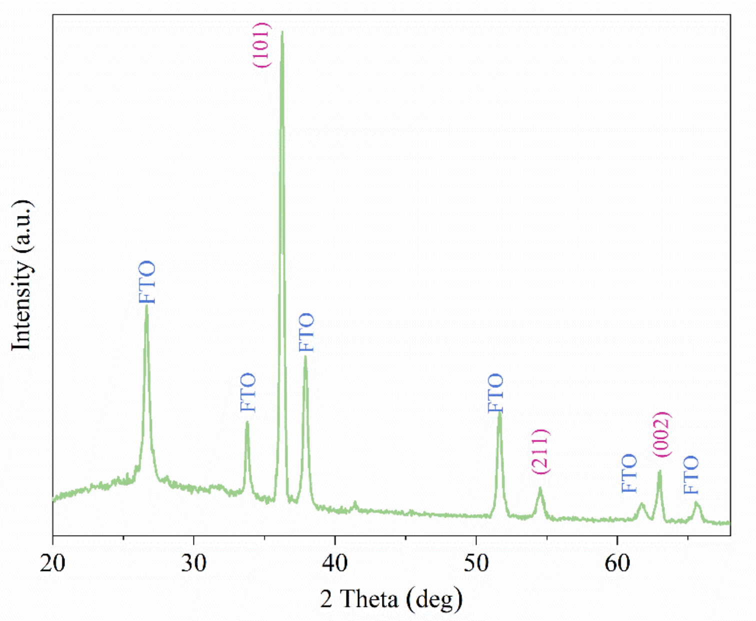

Supplementary Fig. 2 XRD pattern of Pt/TiO_2. No additional peaks were observed after the deposition of Pt atoms, suggesting that no obvious clusters of Pt and its compounds are formed on TiO_2 NRAs.

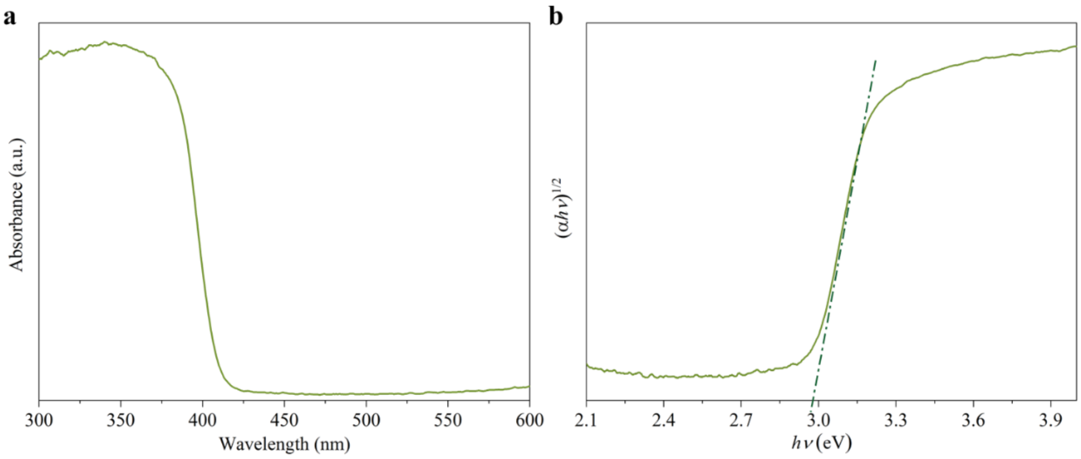

Supplementary Fig. 3 Effects of Pt atoms on the optical adsorption properties. (a) UV–vis absorption spectrum and (b) the corresponding tauc plot of Pt/TiO_2.The deposition of Pt atoms did not induce any obvious changes in the absorption for TiO_2, due to the small loading amount or the tiny size of Pt.

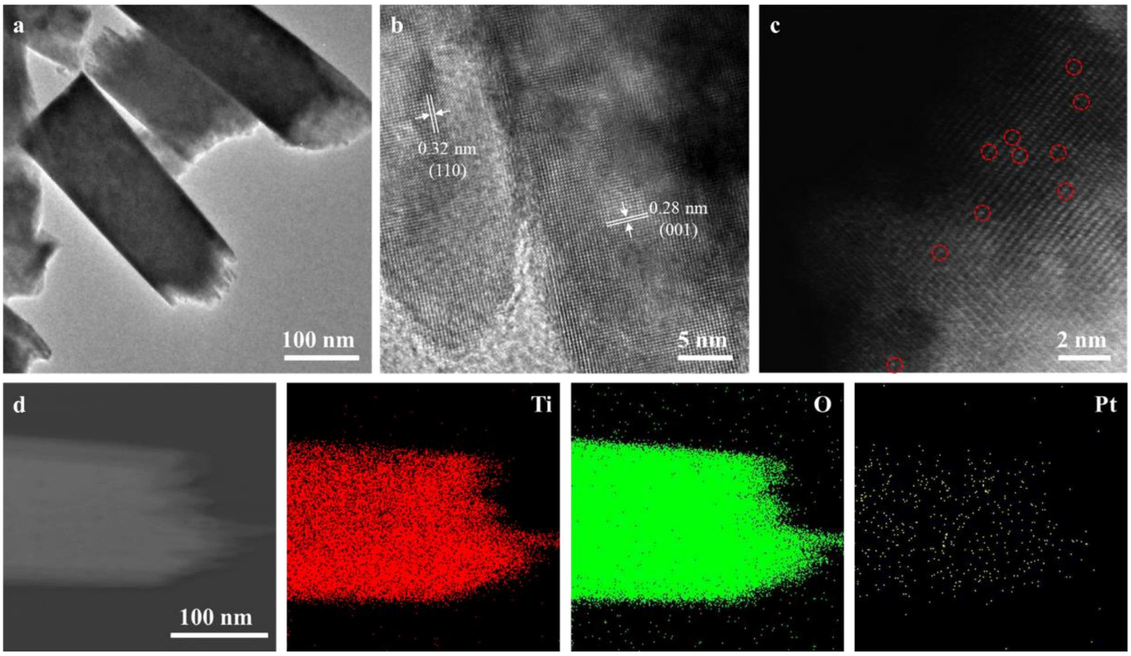

Supplementary Fig. 4 Identification of single atoms on the TiO_2 nanorod. (a) TEM and (b) HR-TEM images of Pt/TiO_2. (c) HAADF-STEM image of Pt/TiO_2 measured from a spherical aberration-corrected TEM. (d) STEM image and the corresponding elemental (Ti, O, Pt) mappings of Pt/TiO_2.

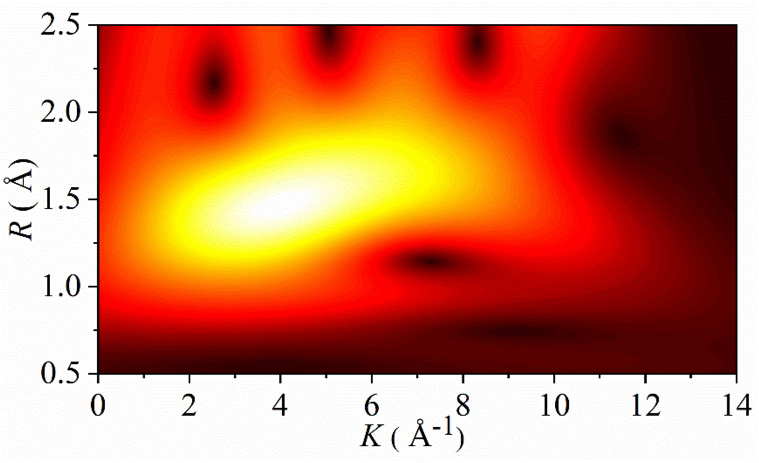

Supplementary Fig. 5 WT-EXAFS of PtO_2 reference at Pt edge. The first coordination shell shows the intensity maximums at ~4 $Å^{-1}$, ascribed to the Pt-O contribution.

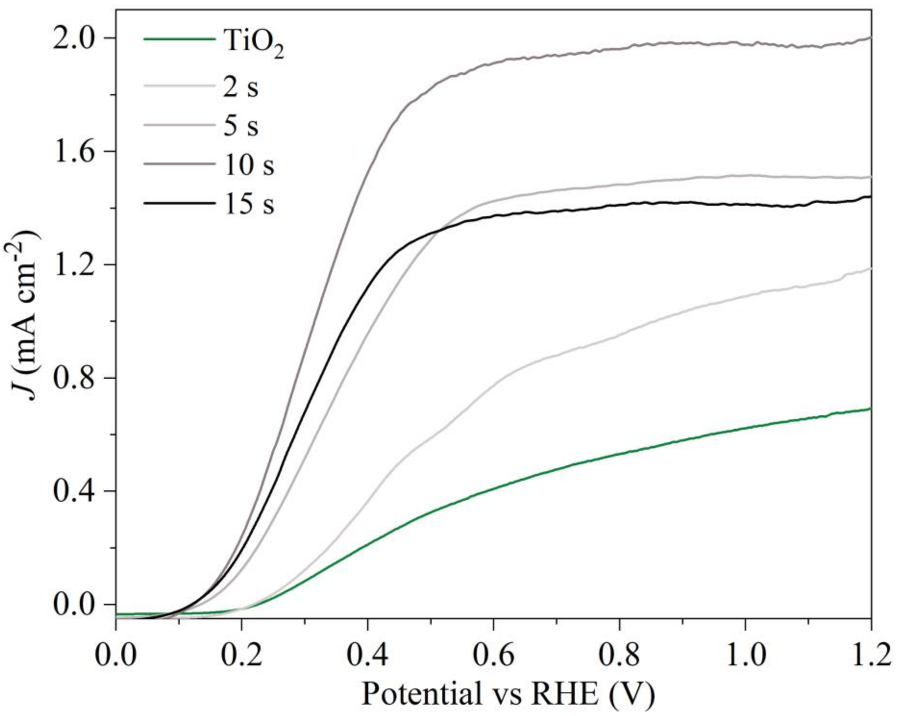

Supplementary Fig. 6 Photocurrent densities of defective TiO_2 photoanodes with different reduction times for glucose oxidation in 1 M KOH with 10 mM glucose under AM 1.5 G, 100 mW cm^{-2} illumination.

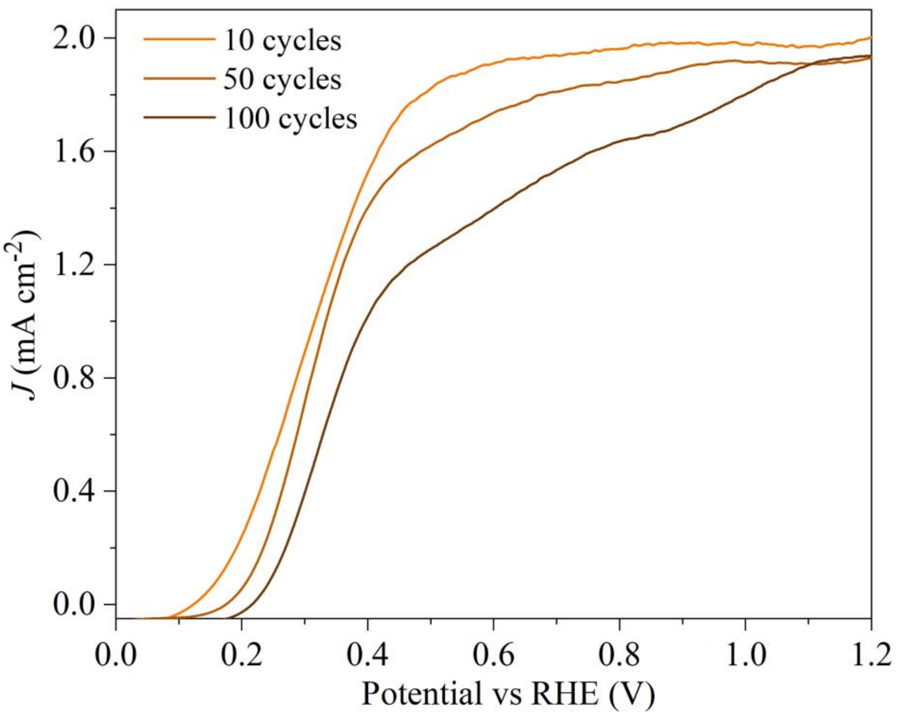

Supplementary Fig. 7 Photocurrent densities of Pt deposited on the def-TiO_2 photoanode with different ALD cycles for glucose oxidation in 1 M KOH with 10 mM glucose under AM 1.5 G, 100 mW cm^{-2} illumination.

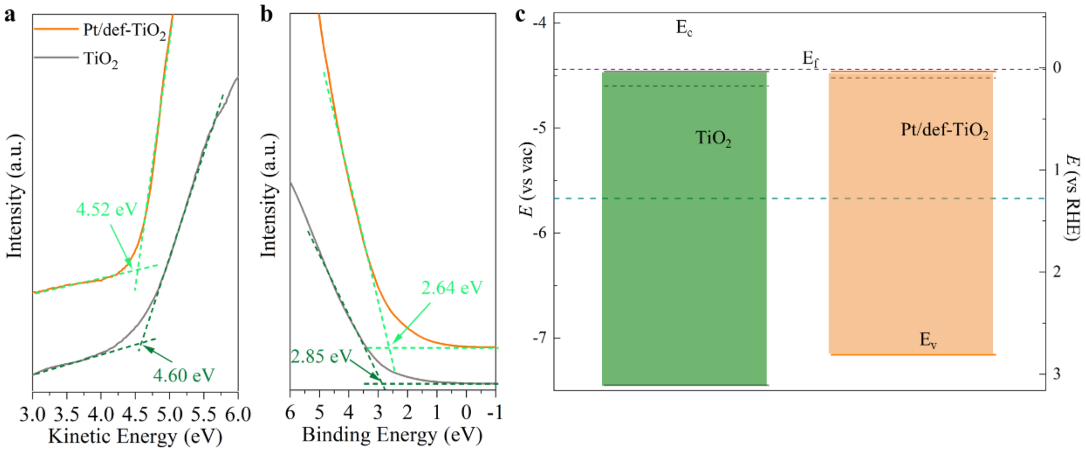

Supplementary Fig. 8 Band alignment between TiO_2 and Pt/def-TiO_2. (a) and (b) UPS spectra of TiO_2 and Pt/def-TiO_2. (c) Band structure diagram of TiO_2 and Pt/def-TiO_2 based on the UPS results.

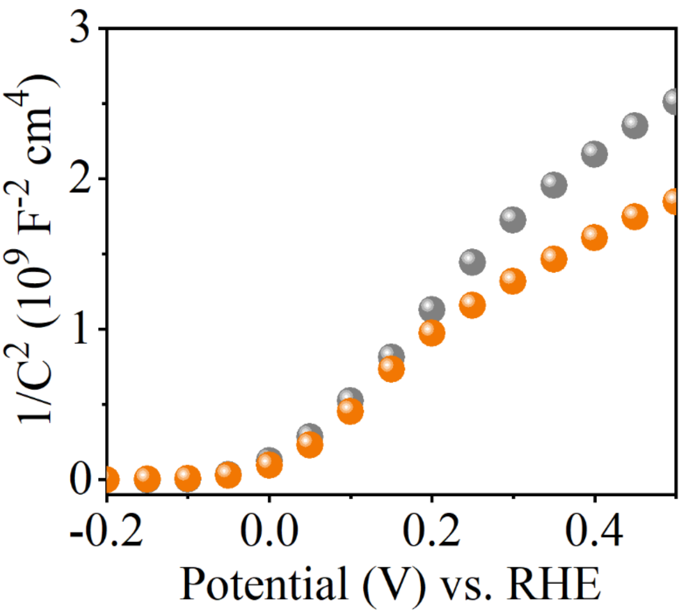

Supplementary Fig. 9 Magnified Mott-Schottky plots of def-TiO_2 and Pt/def-TiO_2 from Fig. 4c.

Supplementary Table 1 Calculated depletion widths in def-TiO_2[a], and Pt/def-TiO_2[b] at different applied voltages.

E (V_{RHE})	**0.3**	**0.4**	**0.5**	**0.6**	**0.7**	**0.8**	**0.9**	**1.0**	**1.1**	**1.2**
r_d (nm)[a]	3.1	3.6	4.1	4.6	4.9	5.3	5.6	6	6.3	6.6
r_d (nm)[b]	2.8	3.4	3.8	4.3	4.6	5.0	5.3	5.6	5.9	6.2

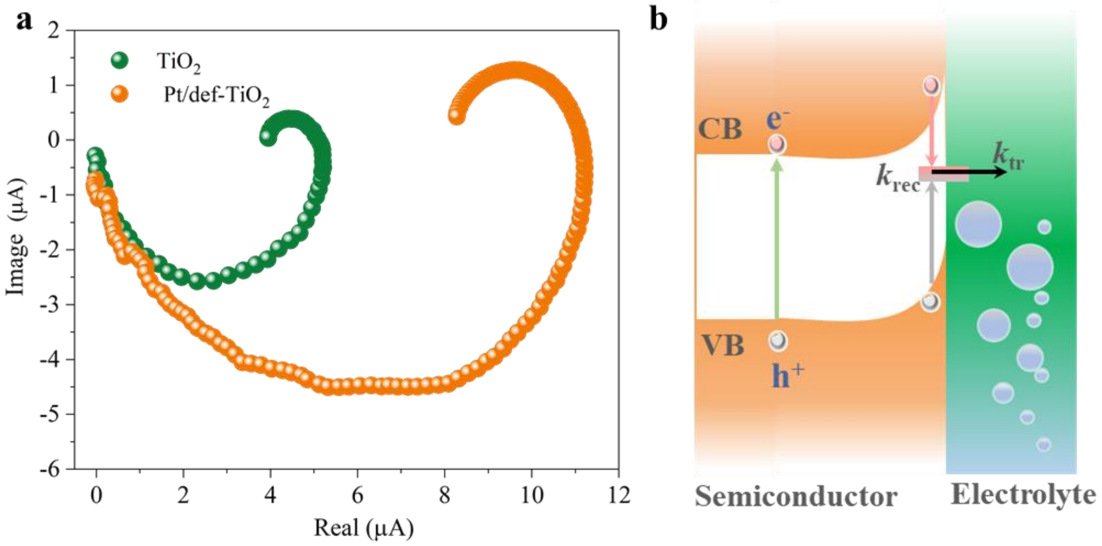

Supplementary Fig. 10 Dynamics of surface charge transfer and recombination. (a) Typical experimental IMPS response measured in 1 M KOH with 0.1 M glucose at 0.8 V_{RHE} for the TiO_2 and Pt/def-TiO_2 photoanodes. (b) Generalized reaction schematics showing the competition between charge transfer and recombination. k_{tr} and k_{rec} are the first-order rate constants for charge transfer and surface recombination, respectively.

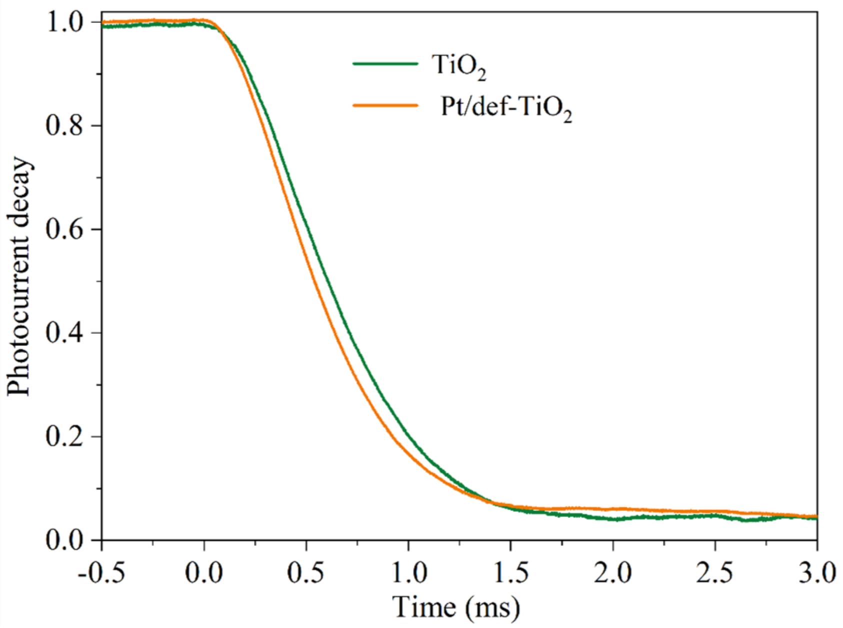

Supplementary Fig. 11 Typical small-perturbation transient photocurrent decay measured in 1 M KOH with 0.1 M glucose at 0.8 V_{RHE} for the TiO_2 and Pt/def-TiO_2 photoanodes.

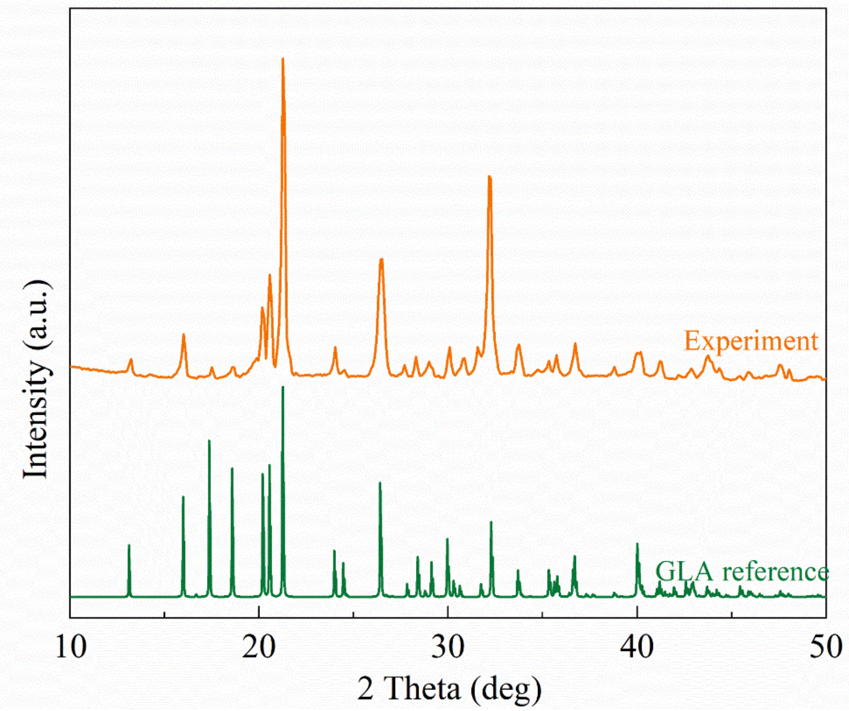

Supplementary Fig. 12. XRD patterns of the products collected from the Pt/def-TiO_2 photoanode and the corresponding GLA reference.

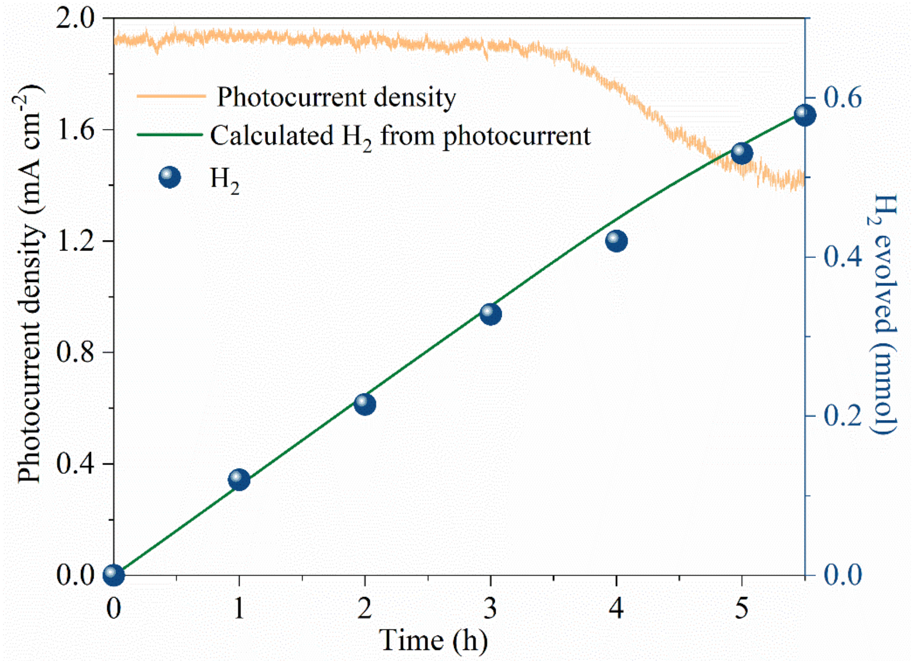

Supplementary Fig. 13 The *J-t* plot of the Pt/def-TiO_2 photoanode at 0.6 V_{RHE} measured in 1 M KOH with 10 mM glucose under AM 1.5 G, 100 mW cm^{-2} illumination, and the corresponding H_2 evolution.

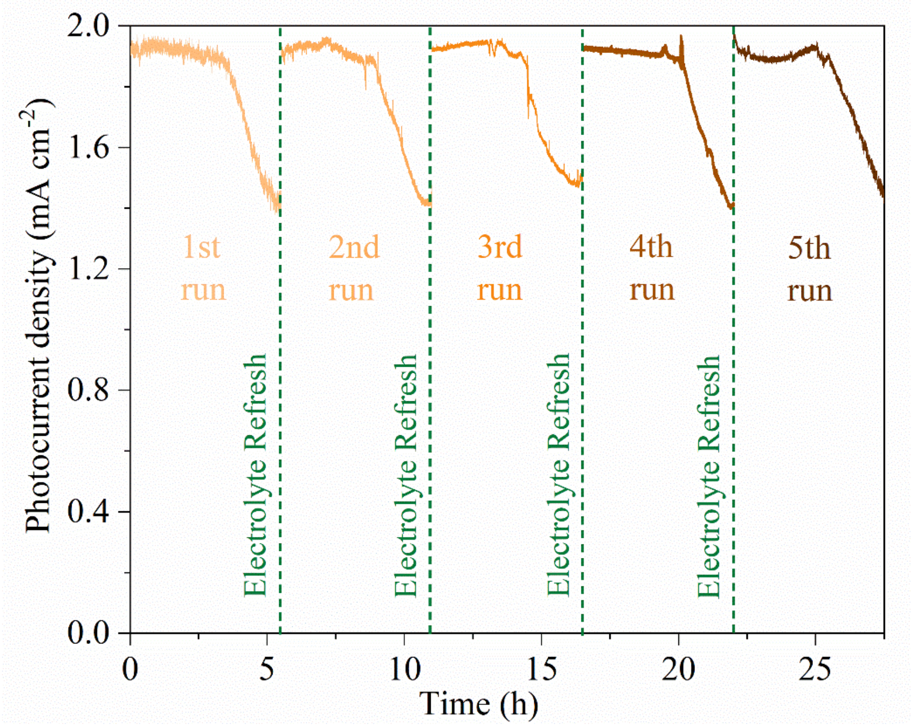

Supplementary Fig. 14 Reproducibility of *J*-*t* plots of the Pt/def-TiO_2 photoanode at 0.6 V_{RHE} from 5 runs measured in 1 M KOH with 10 mM glucose under AM 1.5 G, 100 mW cm^{-2} illumination. The electrolyte was refreshed every 5.5 h.

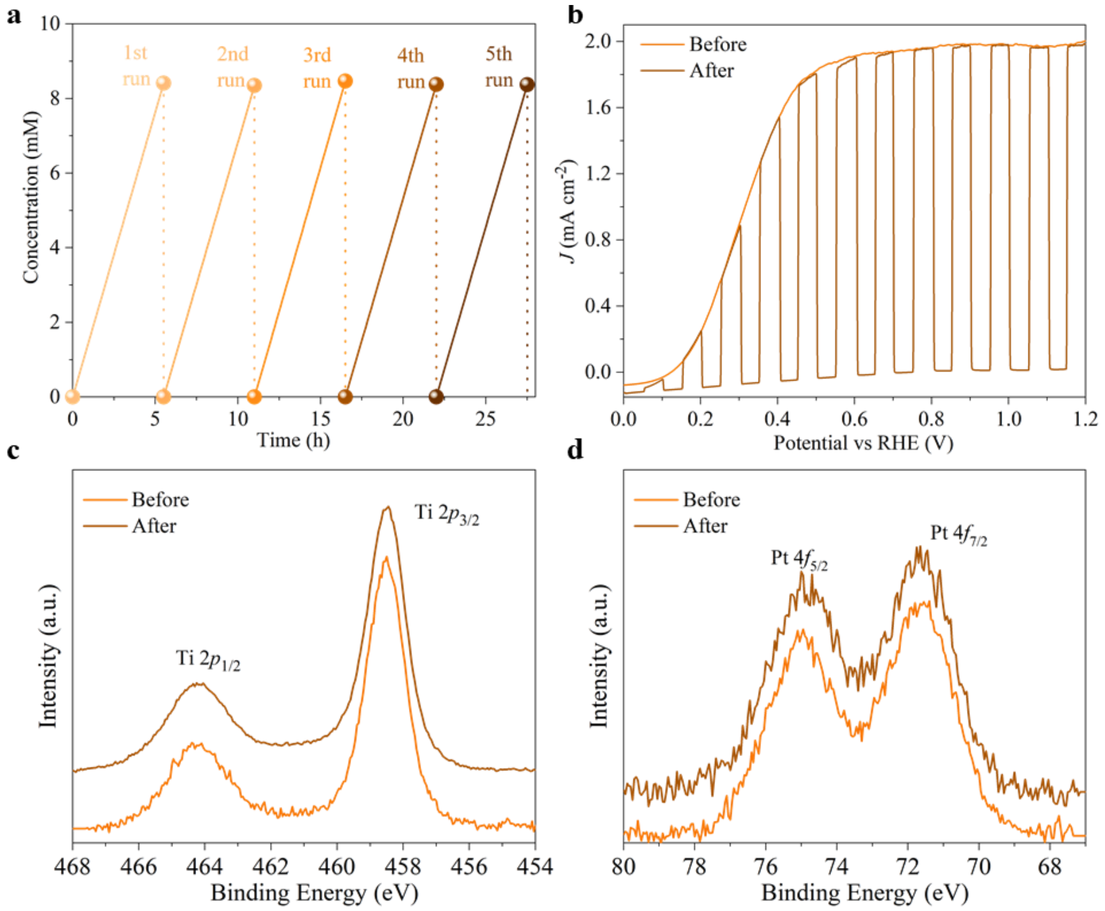

Supplementary Fig. 15 Stability of the Pt/def-TiO_2 photoanode. (a) Results of GLA generation for a repeated PEC reaction sequence over the Pt/def-TiO_2 photoanode under AM1.5G simulated sunlight irradiation. The comparison of (b) photocurrent densities, (c) Ti 2*p* XPS, and (d) Pt 4*f* XPS spectra of the Pt/def-TiO_2 photoanode before and after the 27.5 h PEC test.

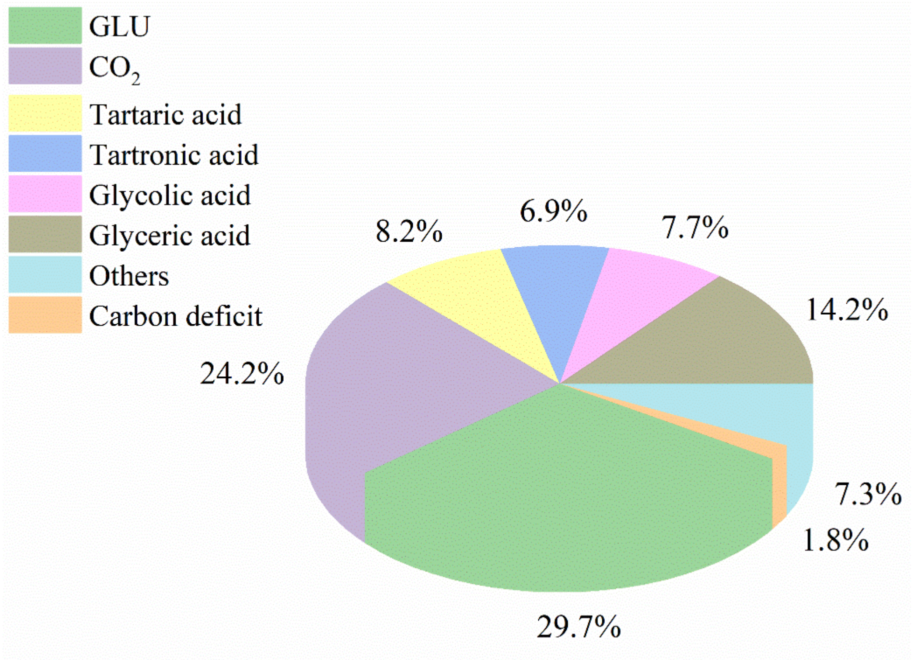

Supplementary Fig. 16 Product distribution of glucose on the TiO_2 photoanode from entry 1 in Table 1. Other acids: oxalic, acetic, and formic acids.

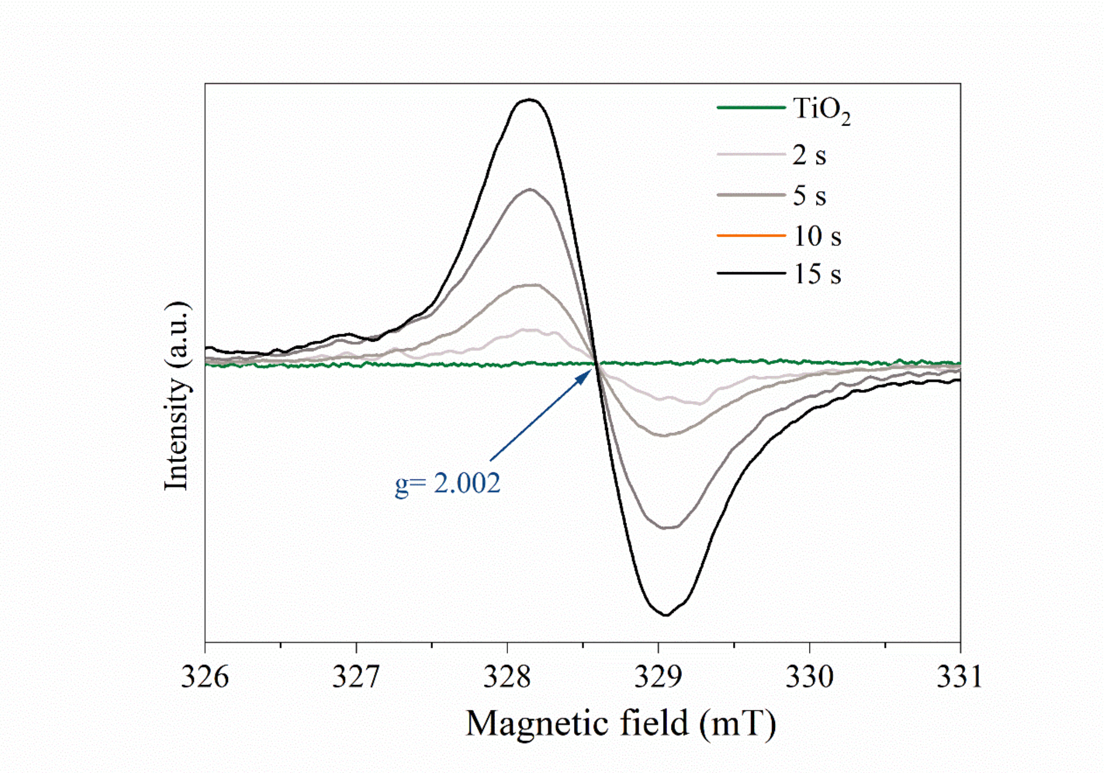

Supplementary Fig. 17 ESR spectra of defective TiO_2 photoanodes with different reduction times. The concentration of the oxygen vacancies in the defective TiO_2 photoanodes increases over the reduction time.

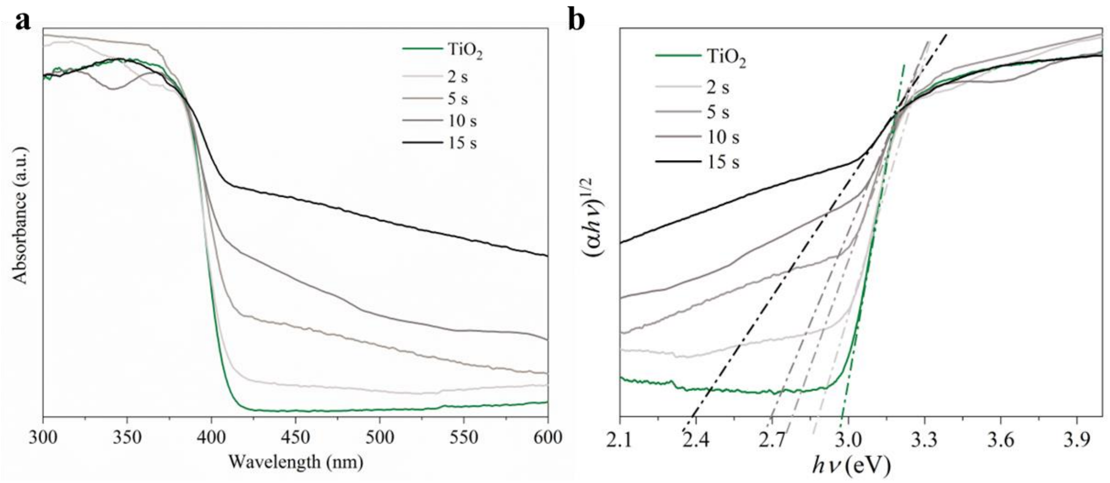

Supplementary Fig. 18 Bandgap changes of the defective for different reduction time. (a) UV–vis absorption spectra and (b) corresponding tauc plots of defective TiO_2 photoanodes with different reduction times. The bandgaps of defective TiO_2 photoanodes decrease over the reduction time, and up-shift VB maximums should account for the decreased bandgaps.

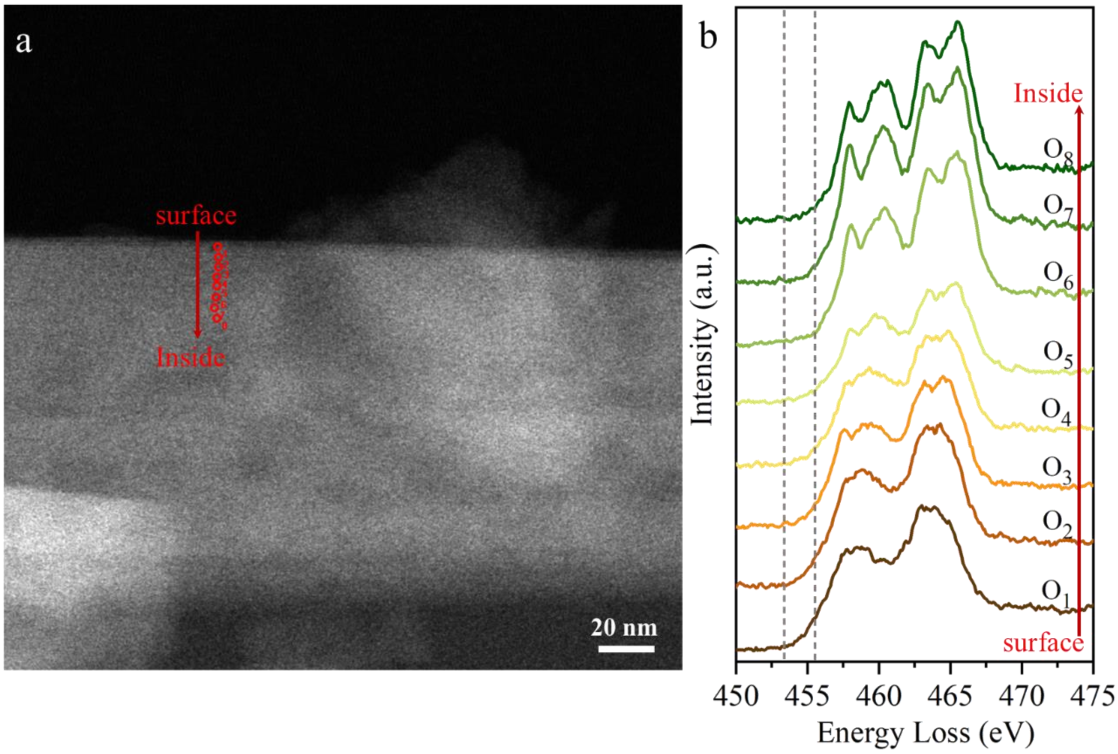

Supplementary Fig. 19 Identification of reduction degree of the def-TiO_2 nanorod. (a) STEM image of a def-TiO_2 nanorod with the probing path shown by the red circles and (b) the corresponding EELS spectra of titanium L2,3 edge. The pitch between the two neighboring spectra is ~3.5 nm.

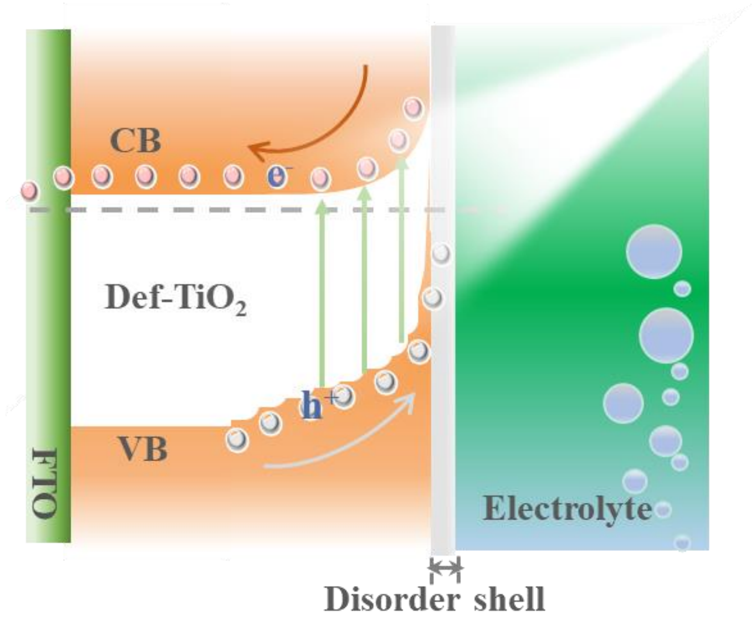

Supplementary Fig. 20 Energy band structure of the def-TiO_2 photoanode and corresponding Energy diagrams for the photo-induced charge transit and transfer.

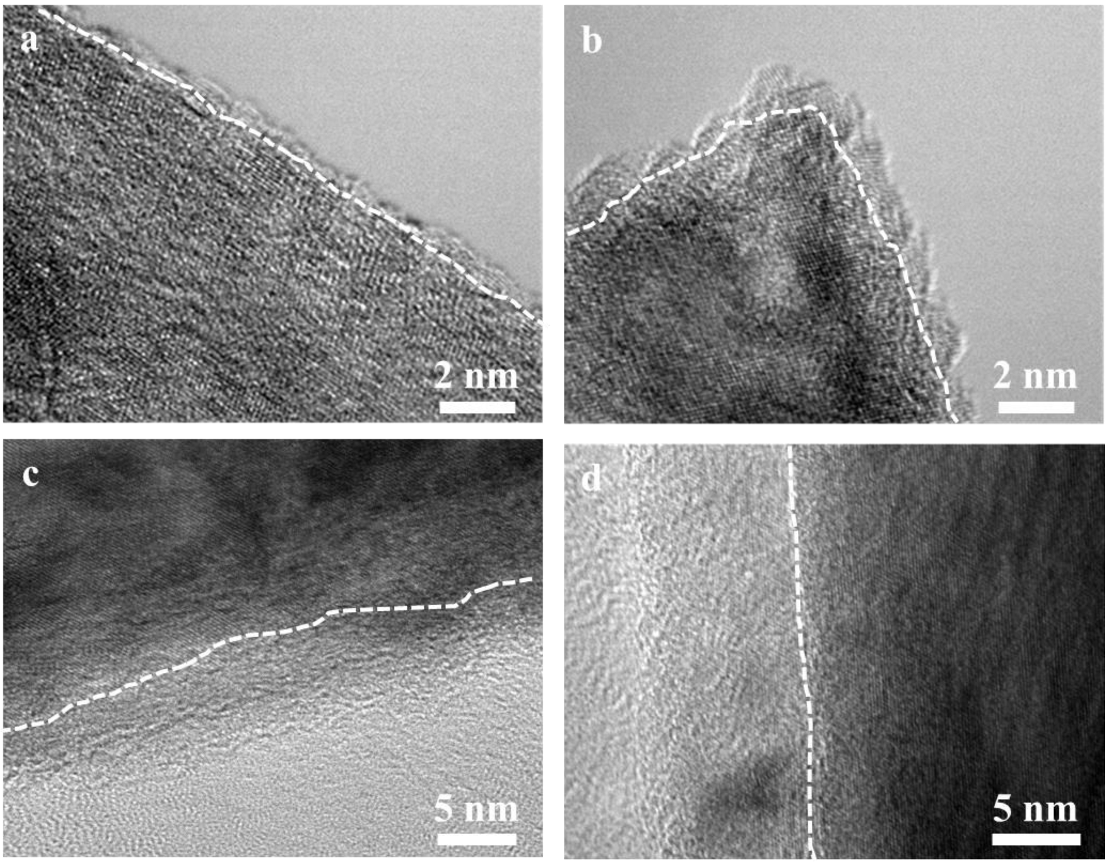

Supplementary Fig. 21 Thicknesses of the disorder for the photoanodes. HRTEM images of defective TiO_2 with (a) 2 s, (b) 5 s, (c) 10 s, and (d) 15 s reduction treatments.

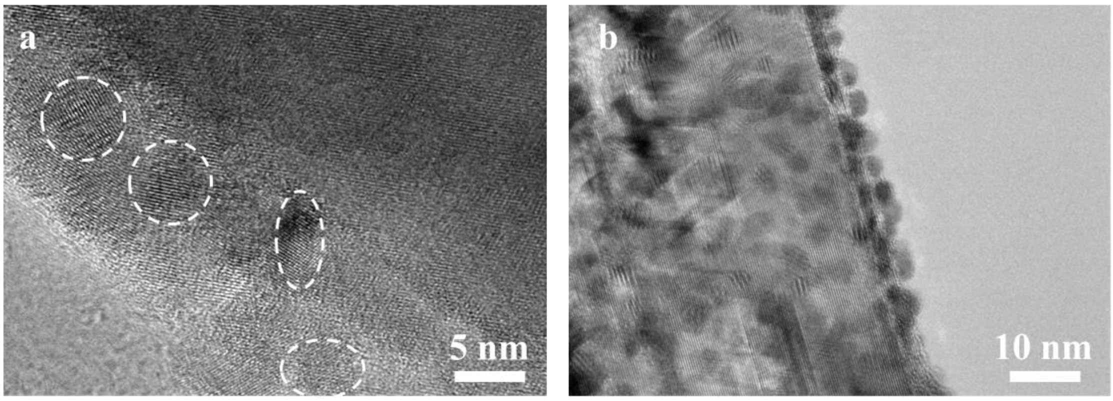

Supplementary Fig. 22 Pt particles size for different ALD cycles. HRTEM images of Pt deposited on defective TiO_2 with (a) 50 and (b) 100 ALD cycles.

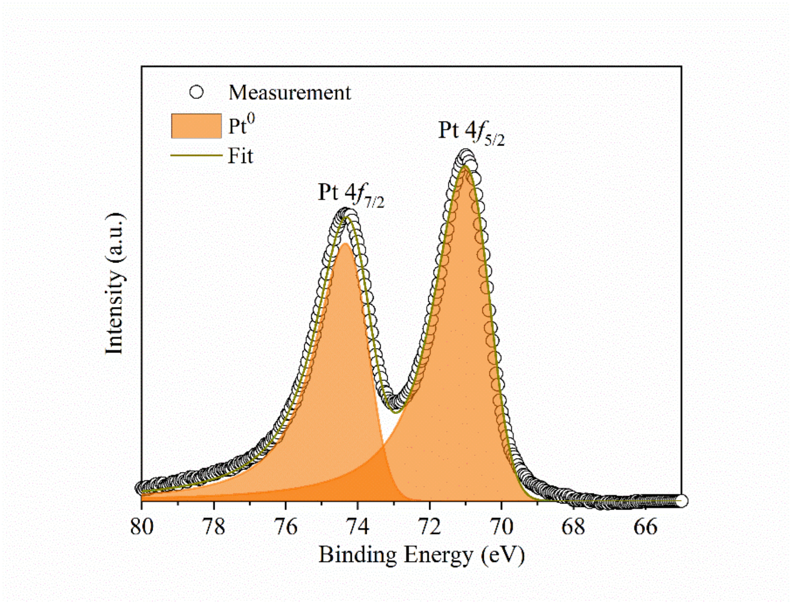

Supplementary Fig. 23 XPS spectrum of Pt 4*f* for Pt particles/def-TiO_2. After 100 ALD cycles, only Pt^0 XPS peaks were observed, suggesting the formation of metallic Pt nanoparticles.

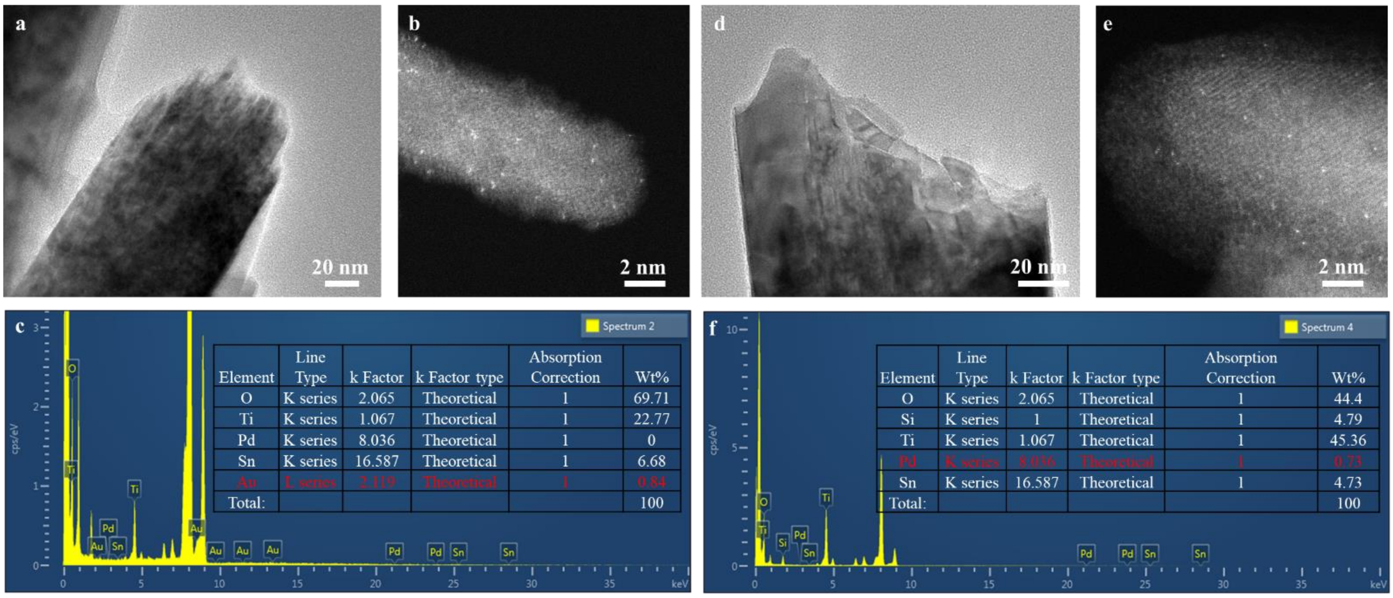

Supplementary Fig. 24 Identification of Pd and Au single atoms on the def-TiO_2 nanorod. (a) TEM image, (b) HAADF-STEM image, and (c) the corresponding EDS spectrum of Au/def-TiO_2. (d) TEM image, (e) HAADF-STEM image, and (f) the corresponding EDS spectrum of Pd/def-TiO_2.

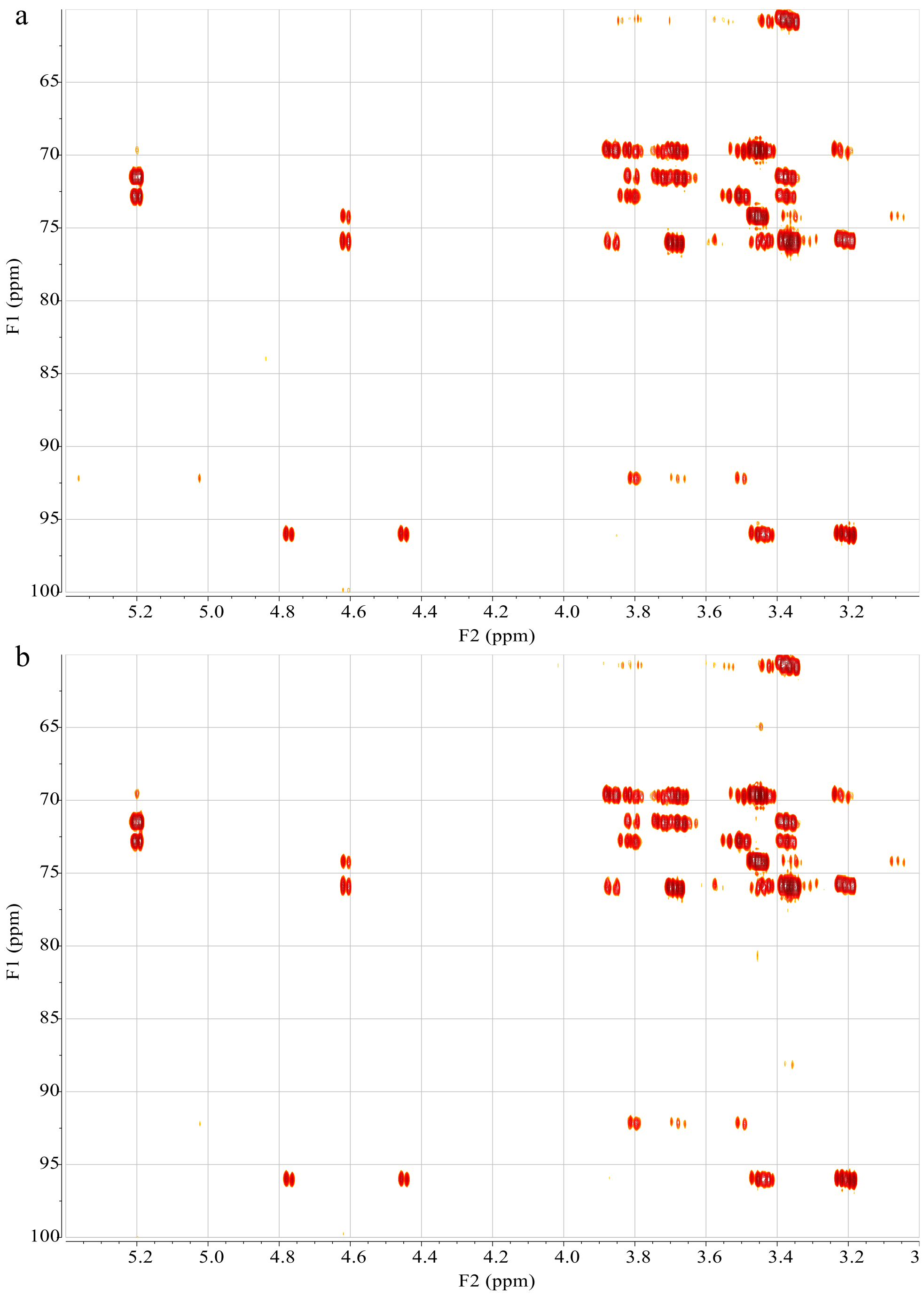

Supplementary Fig. 25 Stability of glucose in alkaline solution. $^{13}C-^{1}H$ HMBC NMR spectra of glucose solutions (a) in its initial form (10 mM glucose in 1 M KOH) and (b) keeping after 10 h.

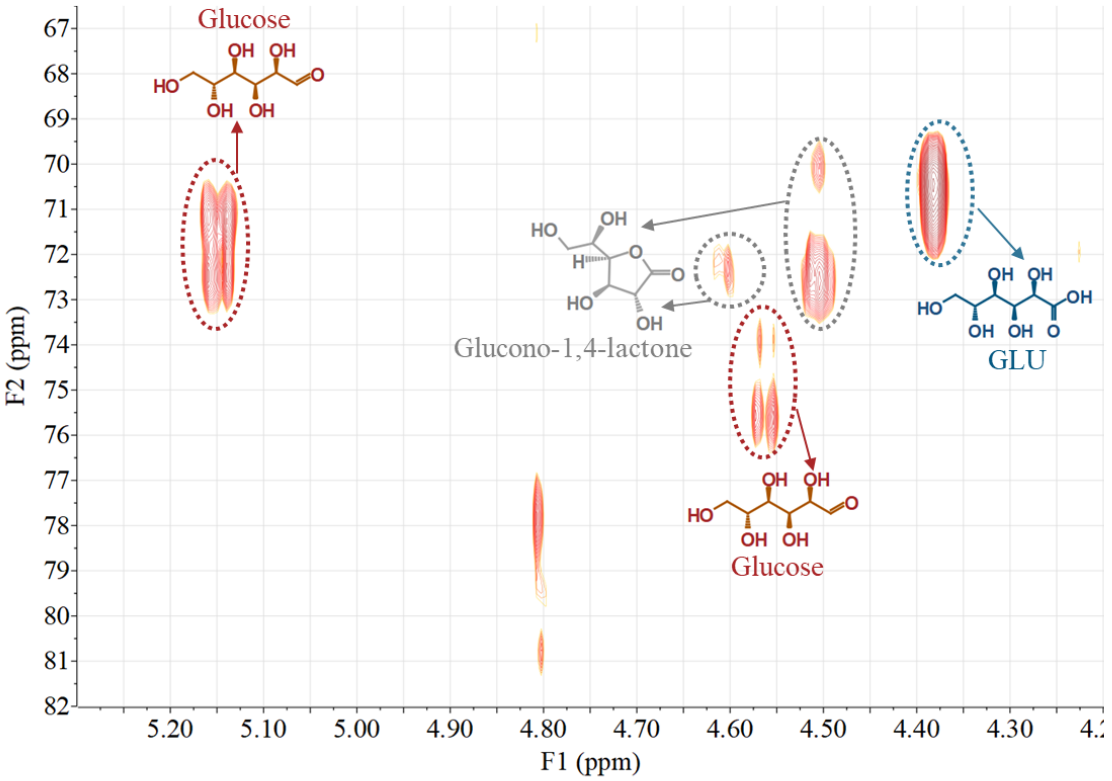

Supplementary Fig. 26 $^{13}C-^{1}H$ HMBC NMR spectrum of the products after 1 h oxidation over the Pt/def-TiO_2 photoanode.

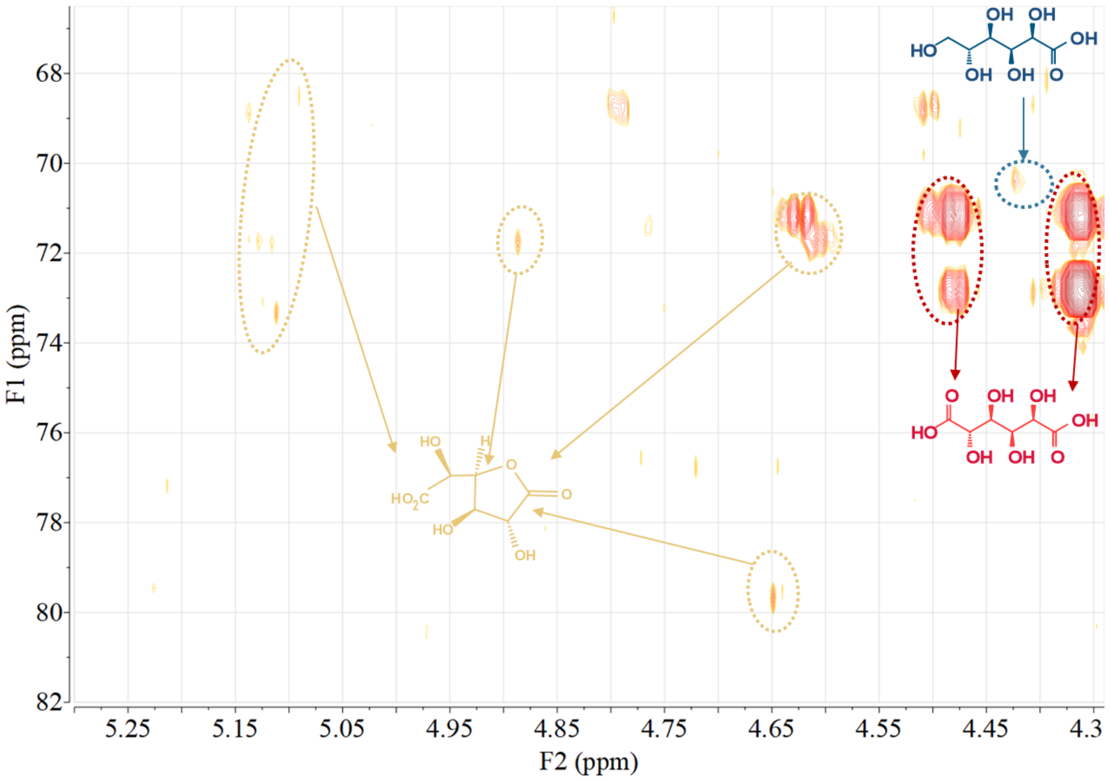

Supplementary Fig. 27 ^{13}C–^{1}H HMBC NMR spectrum of the products after 5.5 h oxidation over the Pt/def-TiO_2 photoanode.

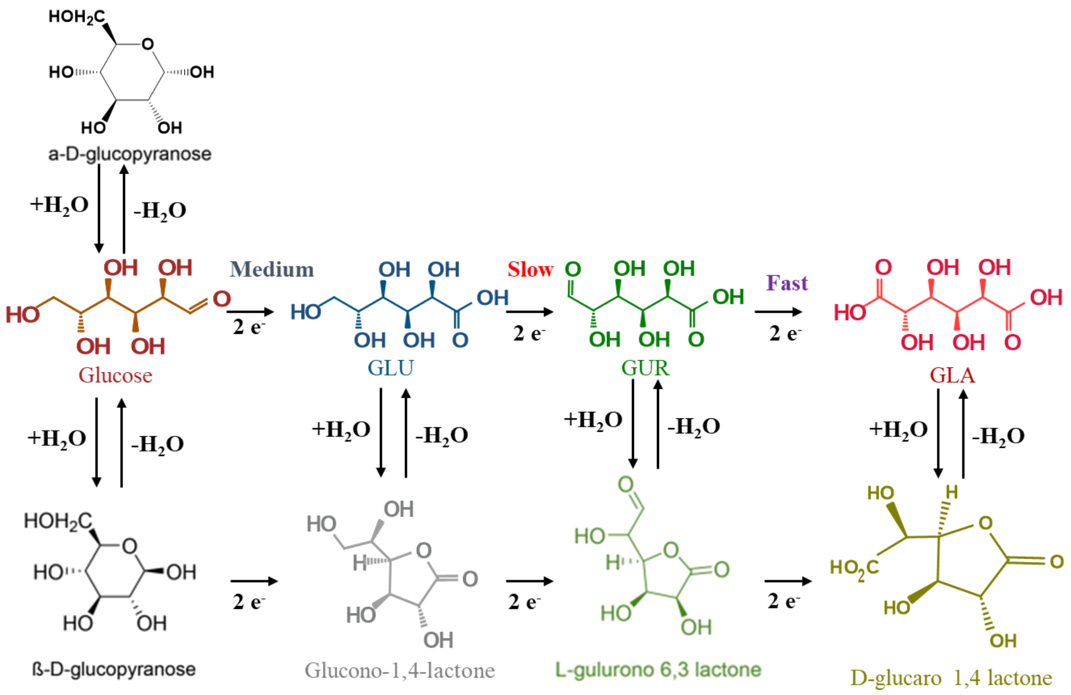

Supplementary Fig. 28 Proposed possible reaction pathway from glucose to GLA. Schematic illustration of the possible pathway for the PEC oxidation of glucose to GLU and GLA over the Pt/def-TiO_2 photoanode.

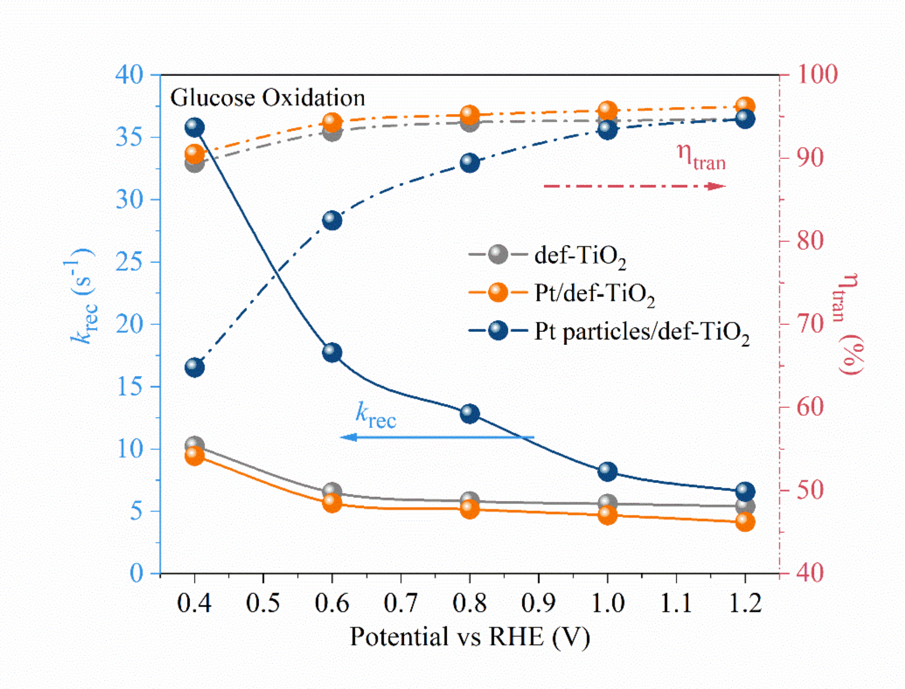

Supplementary Fig. 29 Rate constant of surface recombination (k_{rec}) and surface charge transfer efficiency (η_{tran}) of def-TiO_2, Pt/def-TiO_2, and Pt particles/def-TiO_2 photoanodes for glucose oxidation.

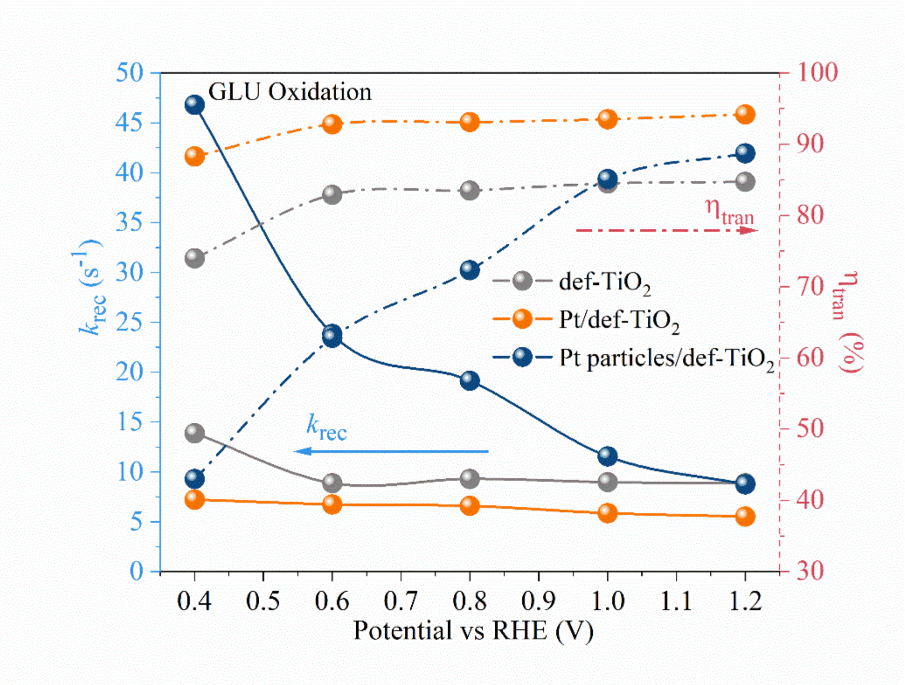

Supplementary Fig. 30 k_{rec} and η_{tran} of def-TiO_2, Pt/def-TiO_2, and Pt particles/def-TiO_2 photoanodes for GLU oxidation.

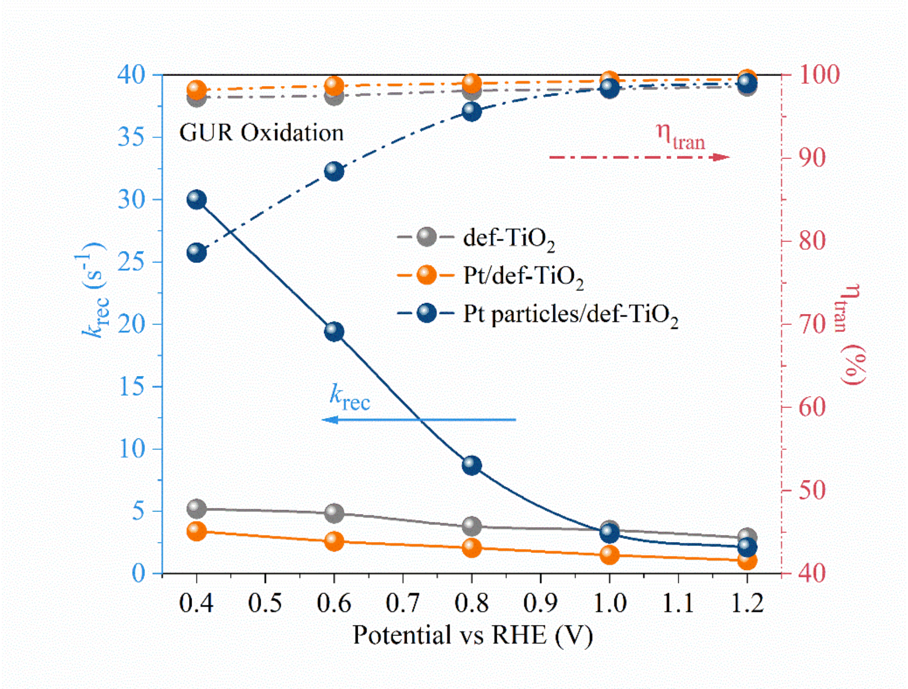

Supplementary Fig. 31 k_{rec} and η_{tran} of def-TiO_2, Pt/def-TiO_2, and Pt particles/def-TiO_2 photoanodes for GUR oxidation.

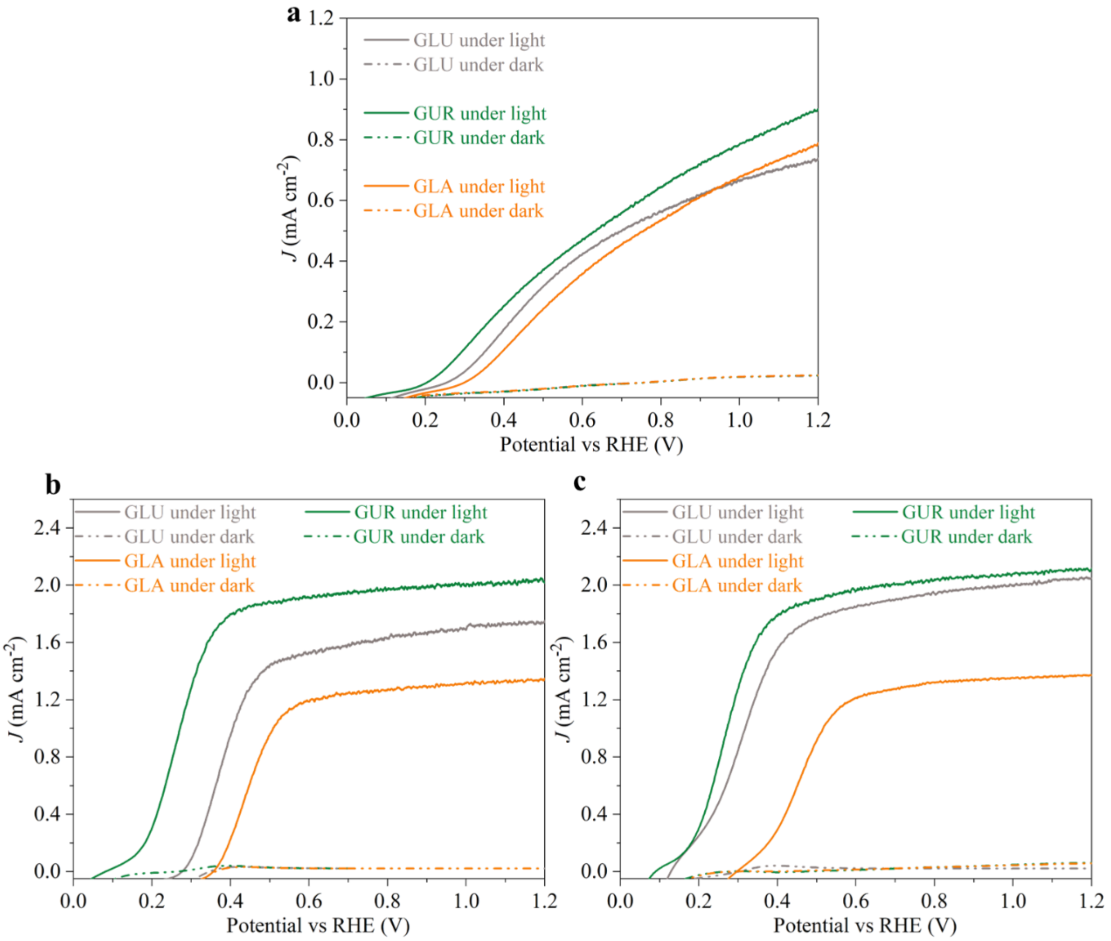

Supplementary Fig. 32 The PEC performance of the Pt/def-TiO_2 photoanodes for GLU, GUR, and GLA oxidation. LSV profiles of (a) the TiO_2, def-TiO_2, (b) and (c) Pt/def-TiO_2 photoanodes for GLU, GUR, and GLA oxidation measured in 1 M KOH with 10 mM GLU, GUR, and GLA, respectively, under AM 1.5 G, 100 mW cm^{-2} illumination and dark.

Supplementary Notes

Supplementary Note 1. Since rutile TiO_2 is a direct bandgap semiconductor,[1] the bandgaps of TiO_2, def-TiO_2, and Pt/def-TiO_2 are estimated to be 2.99, 2.70, and 2.70 eV (Supplementary Fig. 1a), respectively, based on their absorption spectra (Fig. 2e).

Supplementary Note 2. Ultraviolet photoelectron spectroscopy (UPS) measurements of the TiO_2 and Pt/def-TiO_2 photoanodes were carried out to determine their band structures. As shown in Supplementary Fig. 8a**,** the work functions of TiO_2 and Pt/def-TiO_2 are measured to be about 4.60 and 4.52 eV, corresponding to the Fermi levels (E_Fs) of 0.16 and 0.08 V_{RHE}, respectively. The up-shift E_F of Pt/def-TiO_2 is due to the increased electron concentration caused by the oxygen vacancies.[2-4] Based on the valance band information from Supplementary Fig. 8b, the valence band levels (E_Vs) of TiO_2 and Pt/def-TiO_2 are 3.01 and 2.72 V_{RHE}. Since the bandgap of TiO_2 and Pt/def-TiO_2 are 2.99 and 2.70 eV, both the conduction band levels (E_Cs) of TiO_2 and Pt/def-TiO_2 are thus determined to be 0.02 V_{RHE}.

Supplementary Note 3. As shown in Fig. 4c, and Supplementary Fig. 9, all the positive slopes of the samples reveal the n-type semiconductor nature of rutile TiO_2. After reduction treatment, the slope of the def-TiO_2 photoanode is significantly decreased, reflecting the dramatic changes in the carrier density, which can be calculated by using Supplementary Equation (7):[1]

$$N_d=(2/e_0\varepsilon\varepsilon_0)\left[d\left(1/C^2\right)/dV\right]^{-1} \quad (7)$$

where e_0, ε, ε_0, N_d, and $d(1/C^2)/dV$ represent the electron charge, the dielectric constant of rutile TiO_2 (89 for rutile TiO_2), the permittivity of vacuum, the donor density, and the straight slope, respectively. The calculated electron densities of the TiO_2, def-TiO_2, and Pt/def-TiO_2 photoanodes are 5.5×10^{17}, 2.6×10^{20}, and 3.0×10^{20} cm^{-3} separately. Obviously, after the reduction treatment, the electron density in the def-TiO_2 has 3 orders of magnitude enhancement compared to that of TiO_2. This great enhancement is due to the introduction of oxygen vacancies at the disordered shell serving as shallow donors, which can facilitate the charge transport in NRAs.[4] In addition, the flat-band potential (E_{fb}) of the sample can be estimated by extrapolating the plots to $1/C^2 = 0$. Therefore, the E_{fb}s of TiO_2, def-TiO_2, and Pt/def-TiO_2 are estimated to be 0.11, 0.02, and 0.02 V_{RHE}, respectively. The def-TiO_2 photoanode shows a negative shift of E_{fb} compared to the TiO_2 photoanode, consistent with the upward E_F of the def-TiO_2 photoanode observed from the UPS results. The negative shift of E_{fb} will lead to a larger degree of band bending at the surface of def-TiO_2, which will facilitate charge separation at the interface of NRAs and electrolytes.[4,5] Therefore, the induced disorder shell with substantial oxygen vacancies has significant facilitation to the charge carrier dynamics of the NRAs. When compared to the def-TiO_2 photoanodes, the Mott−Schottky changes in the Pt/def-TiO_2 photoanodes are not obvious, suggesting that the further deposition of single-atom Pt has no effects on the charge carrier dynamics of the NRAs.

Supplementary Note 4. The semiconductor depletion region (r_d) could be calculated

using Supplementary Equation (8):[5]

$$r_\mathrm{d} = [\frac{2\varepsilon\varepsilon_0(E-\mathrm{E_{fb}}-\mathrm{k_b}T/\mathrm{q})}{\mathrm{e}N_\mathrm{d}}]^{1/2} \tag{8}$$

After the reduction treatment, the carrier densities of def-TiO_2, and Pt/def-TiO_2 were significantly improved. As a result, the r_d of def-TiO_2, and Pt/def-TiO_2 were limited to 3-7 nm over the test potential range, suggesting a big band bending occurring at the surface of def-TiO_2, and Pt/def-TiO_2. Therefore, fast extraction of holes toward the reactant at the surface/electrolyte junction could happen due to the big band bending, which helped to avoid the internal band-to-band recombination.

Supplementary Note 5. IMPS is a convenient way of measuring the rate constants for charge transfer and recombination and mean electron transport time (τ_d) of an illuminated photoelectrode.[6-9] For the IMPS measurement, all the samples were illuminated with a narrow-band UV light (385 nm, 100 mW cm^{-2}). As shown in Supplementary Fig. 10a, the IMPS shows a typical semicircle in the first quadrant representing the surface charge transfer and recombination process. The τ_t of an illuminated photoelectrode can be calculated from the frequency at the maximum of the semicircle in the fourth quadrant (f_{max4}).[10-15]. It follows that τ_d of an illuminated photoelectrode can be obtained from the optoelectrical admittance plot: $\tau_d \approx 1/2\pi f_{max4}$.

The rate constants k_{tr} and k_{rec} can be extracted from the semicircle in the first quadrant of the IMPS datas using the simple phenomenological theory (Supplementary Fig. 10b).[7,8] At the low-frequency limit, the plot intersects the real axis at the point I_L =

$I_0 k_{tr}/(k_{tr} + k_{rec})$. I_0 is the current density corresponding to the flux of photogenerated minority carriers towards the surface. As the frequency increases, the relaxation in the concentration of photogenerated holes at the semiconductor surface is characterized by f_{max1}, which is the frequency at the maximum of the semicircle in the first quadrant where $2\pi f_{max1} = k_{tr} + k_{rec}$. At the high-frequency limit, the plot intersects the real axis at the point $I_H = I_0$. Thus, based on this information, the k_{tr} and k_{rec} can be calculated.

Supplementary Note 6. Small-perturbation transient photocurrent decay measurements were performed by a similar method to H. J. Snaith *et al*:[16-18] a background light was generated from simulated sunlight (100 mW cm^{-2}) with a narrow-band UV LED light (385 nm) as the perturbation source, controlled by the Zahner Zennium C-IMPS system. The photocurrent dynamics of the PEC cell performed in a three-electrode system were also recorded on the Zahner Zennium C-IMPS system holding a potential difference across a range of 0.6-1.2 V_{RHE}. As shown in Supplementary Fig. 11, the photocurrent dynamics were recorded on the Zahner Zennium C-IMPS system with a 0.5 ms sampling time, a 200 ns response time to an abrupt change in load, and a 10 ms record time. The perturbation light source was set to a suitably low level such that the photocurrent decay kinetics were mono-exponential. For such mono-exponential current decay, while the charge is being collected the charges are also simultaneously recombining within the cell. Therefore, the decay rate constant for the current signal ($1/\tau_{signal}$) is a combination of the decay rate constant for the transport out of the cell ($1/\tau_d$) and the rate constant for the recombination in the cell

($1/\tau_n$) as $1/\tau_{signal} = 1/\tau_{signal} + 1/\tau_{signal}$. Therefore, based on the above-obtained τ_d, the electron lifetime, τ_n, can be achieved.

Supplementary Note 7. The free-glucaric acid can be separated through ion exchange resin, separation by boronic acid affinity gel, and azeotrope drying. Briefly, after the 5.5 h PEC glucose oxidation from the Pt/def-TiO_2 photoanode, the 20 ml electrolyte was first diluted ten times, and then the obtained solution was mixed with 5 g Amberlyst-15 (H^+) ion exchange resin.[19] After 10 mins of ion exchange resin, the collected solution was mixed with boronic acid affinity gel (Affi-Gel boronate gel; Bio-Rad Laboratories, Hercules, CA), and washed with 0.08 M potassium phosphate-0.02 M boric acid buffer (pH 7.0). The GLA was eluted with 0.1 M HCl. The obtained GLA solution was subsequently diluted twenty times with acetonitrile and then the solvent was recovered by rotary evaporation (50 mbar, 22 °C) to yield white powders. The XRD pattern of the collected white powders is consistent with the standard spectra from crystallographic indices derived through single-crystal X-ray diffraction (Supplementary Fig. 12),[19] indicating the purity of the collected GLA.

Supplementary Note 8. As shown in Supplementary Fig. 19, the Ti-L2,3 spectrum taken at 1 nm (O_1) from the surface of the def-TiO_2 nanorod shows two main L3 and L2 peaks with a separation of 5.3 eV and other three shoulder peaks, indicating that the reduction degree in the surface disorder shell is between Ti_4O_7 and TiO_2.[20-24] While the Ti-L2,3 spectrum taken at 28 nm (O_8) from the surface shows the typical EELS spectra

of rutile TiO_2. The gradual shift of the Ti-L2,3 edge towards higher energies from the surface to the inside (from O_1 to O_6) is related to a gradient descent of the Ti^{3+} content. As a result, the def-TiO_2 electrode shows a ~21 nm-thick reduction shell, and the content of oxygen vacancies is gradiently increased from the inside to the surface.

Supplementary Note 9. Supplementary Fig. 25a shows the typical 2D-HMBC NMR of glucose in an aqueous solution.[25] After 10 h, no obvious changes were observed in the 2D-HMBC NMR spectrum (Supplementary Fig. 25b), suggesting no significant spontaneous reaction of glucose.

Supplementary Note 10. The LSV curves for GLU, GUR, and GLA oxidation over the TiO_2, def-TiO_2, and Pt/def-TiO_2 photoanodes were tested with and without illumination in the electrolytes of 1 M KOH with 10 mM GLU, GUR, and GLA, respectively. As shown in Supplementary Fig. 32, the dark currents for the GLU, GUR, and GLA oxidations over all the photoanodes can be neglected compared to their corresponding photocurrents, suggesting that the GLU, GUR, and GLA oxidations over all the photoanodes are PEC reactions. Besides, the photocurrent densities of all the photoanodes follow this sequence: $J_{GUR}> J_{GLU}> J_{GLA}$, further revealing the fast kinetics for GUR oxidation. The TiO_2 photoanode shows much smaller differences in the J_{GUR}, J_{GLU}, and J_{GLA} than the def-TiO_2, and Pt/def-TiO_2 photoanodes, probably due to that the cleavage of C-C bonds is the main reaction on the TiO_2 photoanode. With the cleavage of C-C bonds suppressed, the def-TiO_2 photoanode shows distinctly different photocurrents for the GLU, GUR, and GLA oxidations. The J_{GLU} is obviously smaller

than $J_{glucose}$ (Fig. 4a) and J_{GUR} (Supplementary Fig. 32b), also confirming the rate-limiting step of GLU oxidation on the def-TiO_2 photoanode. The further deposition of Pt SAs significantly promotes GLU oxidation (Supplementary Fig. 32c), accelerating the conversion of GLA from GLU. Furthermore, sluggish kinetics for GLA oxidation are observed on the Pt/def-TiO_2 photoanode. The accelerated GLU oxidation and sluggish GLA oxidation consequently result in a high selectivity of glucose to GLA over the Pt/def-TiO_2 photoanode.

Supplementary References

1 Tian, Z. *et al.* Efficient charge separation of in-situ Nb-doped TiO_2 nanowires for photoelectrochemical water-splitting. *ChemistrySelect* **2**, 2822-2827 (2017).

2 Wang, Z. *et al.* H-doped black titania with very high solar absorption and excellent photocatalysis enhanced by localized surface plasmon resonance. *Adv. Funct. Mater.* **23**, 5444-5450 (2013).

3 Tian, Z. *et al.* Hydrogen plasma reduced black TiO_2-B nanowires for enhanced photoelectrochemical water-splitting. *J. Power Sources* **325**, 697-705 (2016).

4 Tian, Z. *et al.* Highly conductive cable-like bicomponent titania photoanode approaching limitation of electron and hole collection. *Adv. Funct. Mater.* **36**, 1803328 (2018).

5 Zhang, K. *et al.* Overcoming charge collection limitation at solid/liquid interface by a controllable crystal deficient overlayer. *Adv. Energy Mater.* **7**, 1600923 (2017).

6 Gao, Y. & Hamann, T. W. Quantitative hole collection for photoelectrochemical water oxidation with $CuWO_4$. *ChemComm.* **53**, 1285-1288 (2017).

7 Cachet, H. & Sutter, E. M. M. Kinetics of water oxidation at TiO_2 nanotube arrays at different pH domains investigated by electrochemical and light-modulated impedance spectroscopy. *J. Phys. Chem. C* **119**, 25548-25558 (2015).

8 Peter, L. M. Energetics and kinetics of light-driven oxygen evolution at semiconductor electrodes: the example of hematite. *J. Solid State Electrochem.* **17**, 315-326 (2013).

9 Ponomarev, E. A. & Peter, L. M. A Comparison of intensity-modulated

photocurrent spectroscopy and photoelectrochemical impedance spectroscopy in a study of photoelectrochemical hydrogen evolution at P-Inp. *J. Electroanal. Chem.* **397**, 45-52 (1995).

10 de Jongh, P. E. & Vanmaekelbergh, D. Trap-limited electronic transport in assemblies of nanometer-size TiO_2 particles. *Phys. Rev. Lett.* **77**, 3427-3430 (1996).

11 de Jongh, P. E. & Vanmaekelbergh, D. Investigation of the electronic transport properties of nanocrystalline particulate TiO_2 electrodes by intensity-modulated photocurrent spectroscopy. *J. Phys. Chem. B* **101**, 2716-2722 (1997).

12 de Jongh, P. E., Meulenkamp, E. A., Vanmaekelbergh, D. & Kelly, J. J. Charge carrier dynamics in illuminated, particulate ZnO electrodes. *J. Phys. Chem. B* **104**, 7686-7693 (2000).

13 Vanmaekelbergh, D., Marin, F. I. & vandeLagemaat, J. Transport of photogenerated charge carriers through crystalline GaP networks investigated by intensity modulated photocurrent spectroscopy. *Berichte Der Bunsen-Gesellschaft- Phys. Chem. Chem. Phys.* **100**, 616-626 (1996).

14 Chen, H., Wei, Z., Yan, K., Bai, Y. & Yang, S. Unveiling two electron-transport modes in oxygen-deficient TiO_2 nanowires and their influence on photoelectrochemical operation. *J. Phys. Chem. Lett.* **5**, 2890-2896 (2014).

15 Lynch, R. P., Ghicov, A. & Schmuki, P. A Photo-electrochemical investigation of self-organized TiO_2 nanotubes. *J. Electrochem. Soc.* **157**, G76-G84 (2010).

16 Snaith, H. J. *et al.* Charge collection and pore filling in solid-state dye-sensitized solar cells. *Nanotechnology* **19**, 424003 (2008).

17 Docampo, P. *et al.* Control of solid-state dye-sensitized solar cell performance by block-copolymer-directed TiO_2 synthesis. *Adv. Funct. Mater.* **20**, 1787-1796 (2010).

18 Lee, M. M., Teuscher, J., Miyasaka, T., Murakami, T. N. & Snaith, H. J. Efficient hybrid solar cells based on meso-superstructured organometal halide perovskites. *Science* **338**, 643-647 (2012).

19 Armstrong, R. D., Kariuki, B. M., Knight, D. W. & Hutchings, G. J. How to synthesise high purity, crystalline d-glucaric acid selectively. *Eur. J. Org. Chem.* **2017**, 6811-6814 (2017).

20 Bertoni, G. *et al.* Quantification of crystalline and amorphous content in porous samples from electron energy loss spectroscopy. *Ultramicroscopy* **106**, 630-635 (2006).

21 Gao, Q. *et al.* Direct evidence of lithium-induced atomic ordering in amorphous TiO_2 nanotubes. *Chem. Mater.* **26**, 1660-1669 (2014).

22 Tian, M. *et al.* Structure and formation mechanism of black TiO_2 nanoparticles. *Acs Nano* **9**, 10482-10488 (2015).

23 Azor-Lafarga, A. *et al.* Modified synthesis strategies for the stabilization of low n Ti_nO_{2n-1} magneli phases. *Chem Rec.* **18**, 1105-1113 (2018).

24 Stoyanov, E., Langenhorst, F. & Steinle-Neumann, G. The effect of valence state and site geometry on Ti $L_{3,2}$ and O K electron energy-loss spectra of Ti_xO_y phases. *Am. Mineral.* **92**, 577-586 (2007).

25 Armstrong, R. D., Hirayama, J., Knight, D. W. & Hutchings, G. J. Quantitative

determination of Pt-catalyzed d-glucose oxidation products using 2D NMR. *ACS Catal.* **9**, 325-335 (2018).
